# Epithelial-Mesenchymal Transition with Malignant Transformation Leading Multiple Metastasis from Disseminated Peritoneal Leiomyomatosis

**DOI:** 10.3390/jcm7080207

**Published:** 2018-08-09

**Authors:** Hsiao-Chen Chiu, Meng-Yu Wu, Chao-Hsu Li, Su-Cheng Huang, Giou-Teng Yiang, Hsuan-Shang Yen, Wei-Lin Liu, Chia-Jung Li, Woei-Yau Kao

**Affiliations:** 1 Department of Obstetrics and Gynecology, Taipei Tzu Chi Hospital, Buddhist Tzu Chi Medical Foundation, Taipei 231, Taiwan; 97311141@gms.tcu.edu.tw (H.-C.C.); schuang@tzuchi.com.tw (S.-C.H.); julia791212@gmail.com (H.-S.Y.); 2 Department of Obstetrics and Gynecology, School of Medicine, Tzu Chi University, Hualien 970, Taiwan; 3 Department of Emergency Medicine, Taipei Tzu Chi Hospital, Buddhist Tzu Chi Medical Foundation, New Taipei 231, Taiwan; skyshangrila@gmail.com (M.-Y.W.); gtyiang@gmail.com (G.-T.Y.); 4 Department of Emergency Medicine, School of Medicine, Tzu Chi University, Hualien 970, Taiwan; 5 Department of Surgery, Taipei Tzu Chi Hospital, Buddhist Tzu Chi Medical Foundation, Taipei 231, Taiwan; xd710212@tzuchi.com.tw; 6 Department of Radiation Oncology, Show Chwan Memorial Hospital, Changhua 500, Taiwan; 7 Research Assistant Center, Show Chwan Memorial Hospital, Changhua 500, Taiwan; 8 Department of Hematology and Oncology, Taipei Tzu Chi Hospital, Buddhist Tzu Chi Medical Foundation, Taipei 231, Taiwan

**Keywords:** leiomyomatosis, peritonealis disseminata, leiomyosarcoma, sarcomatous transformation

## Abstract

Disseminated peritoneal leiomyomatosis (DPL) is a rare condition that is characterized by the presence of multiple subperitoneal or peritoneal smooth muscle nodules of varying sizes on the omentum and peritoneal surfaces, grossly mimicking disseminated carcinoma. DPL usually develops in premenopausal women with a benign course, and it is often found incidentally during abdominal surgery. Malignant transformation is a rare clinical course of DPL. Only a few studies have focused on DPL transformation into a leiomyosarcoma. Herein, we describe the case of a 61-year-old woman with a history of recurrent leiomyoma of the uterus who presented with intermittent progressive abdominal pain. The imaging study revealed a huge heterogeneous density mass in the pelvic region with pulmonary and hepatic metastases. Exploratory laparotomy and debulking surgery were performed, and showed the coexistence of DPL and leiomyosarcoma. She died approximately one month after the diagnosis because of rapid progression of pleural effusion due to malignancy. This case highlights the clinical features of DPL and its malignant transformation and metastasis so physicians can make an early diagnosis and provide timely management.

## 1. Introduction

DPL is an extremely rare condition that is characterized by the presence of multiple smooth muscle, fibroblastic, and myofibroblastic nodules on the peritoneal surface of the pelvis and abdominal cavity [1]. The true prevalence of DPL is unclear because most individuals with DPL remain asymptomatic [2]. DPL usually occurs in premenopausal women and has a benign clinical course. Thus, it is very rare for DPL to become malignant. In previous literature, only 10 cases of DPL were associated with malignant transformation [3,4,5,6,7,8,9,10,11,12]. In advanced malignant transformation, the distant metastasis may progress rapidly in patients with DPL. Therefore, early diagnosis, timely surgical intervention, and appropriate chemotherapy are very important in order for physicians to control the progression of malignant transformation and metastasis. We describe the clinical features of DPL and highlight its malignant transformation and metastasis in an elderly woman.

## 2. Case Presentation

This case study was approved by the Institutional Review Board (07-CR-085) of Taipei Tzu Chi Hospital, Buddhist Tzu Chi Medical Foundation.

A 61-year-old G2P1AA1 woman presented with intermittent, progressive lower abdominal pain for three years. She had a medical history of hypertension that was controlled with medication. In 1993, she was diagnosed as having a uterine leiomyoma with leiomyomatosis of retroperitoneal tumors. Because of her desire to bear children in the future, she underwent myomectomy at that time. Subsequently, recurrent DPL developed two years later due to an enlarged palpable mass in the abdomen with intermittent abdominal pain. She underwent abdominal hysterectomy with left-sided oophorectomy and right ovary wedge resection in 1996. Results of the pathological report showed a borderline mucinous ovarian tumor of the left ovary. In 1998, recurrent DPL with left ureter involvement was noted, and the third operation involving debulking surgery and reteroneocystostomy was performed. Results of that pathological report showed no significant malignancy. She was regularly followed up at gynecologic clinics. In 1999, a 9 × 9-cm^2^ palpable pelvic mass was observed, and recurrent leiomyomatosis was suspected. A fourth laparotomy for tumor excision was suggested to the patient, but she refused. Hormone therapy with medroxyprogesterone (5-mg tablet daily) was performed until 2014 because of the progression of a thyroid nodular goiter and hypertension. After stopping hormone therapy, she reported intermittent, progressive lower abdominal pain, and a growing palpable mass was observed in the abdomen.

**Figure 1:**
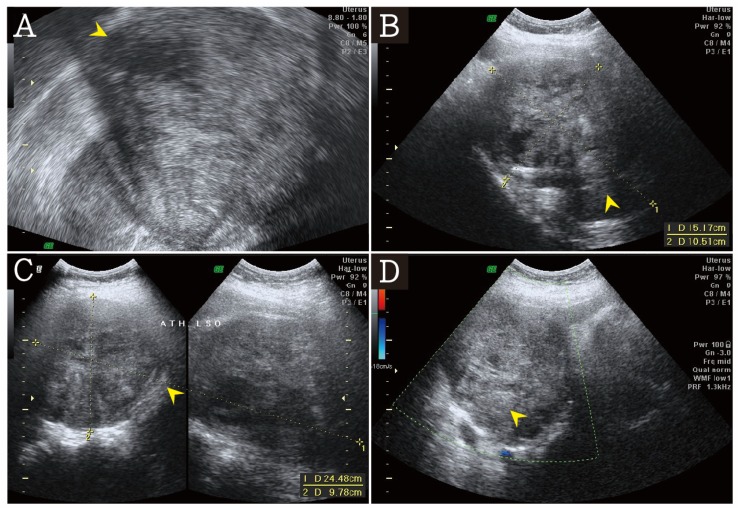
(**A**) The transvaginal ultrasound revealed a huge heterogeneous echogenicity mass (arrow head). (**B**,**C**) The heterogeneous mass was about 15.17 × 24.48 × 10.51 cm^3^ (arrow head). (**D**) In doppler color flow mapping, no significant blood flow was noted in the heterogeneous mass (arrow head).

On admission, her temperature was 36.6 °C, blood pressure was 123/69 mmHg, heart rate was 83 beats/min, body weight was 59.8 kg, body height was 155.5 cm, and body mass index was 24.7 kg/m^2^. On pelvic examination, a huge tumor was palpable in the pelvis. Transvaginal ultrasonography showed huge solid tumors measuring about 15.17 × 24.48 × 10.51 cm^3^ in the pelvic field without free fluid in the cul-de-sac region (Figure 1A–D).

**Figure 2:**
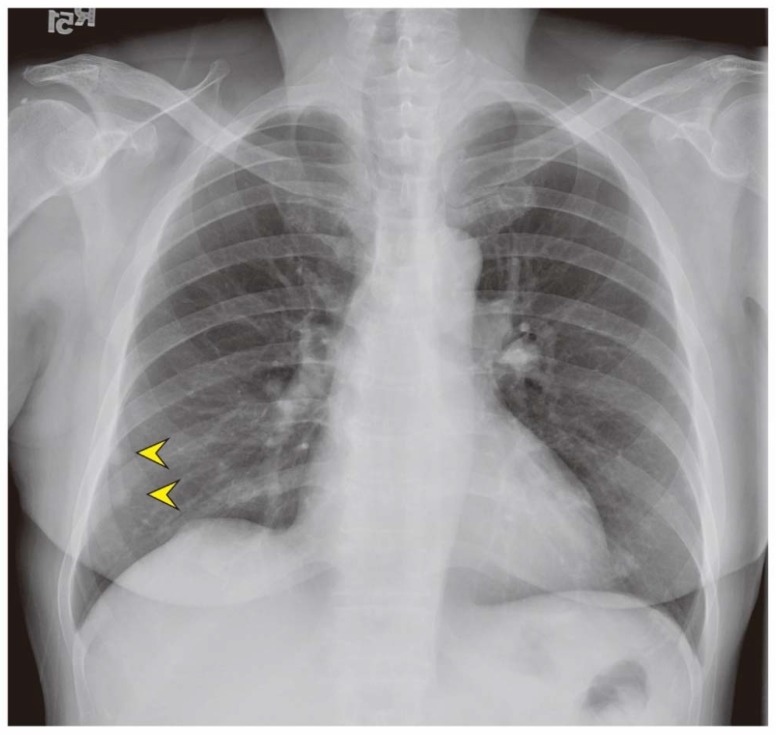
The chest X-ray revealed two homogeneous pulmonary nodules at right lower lung field (arrow head).

The laboratory evaluation showed the following: white blood cell count, 13,520 µL; hemoglobin level, 7.8 g/dL; fibrin degradation product level, >10,000 ng/mL; follicle-stimulating hormone level, 15.98 mIU/mL; progesterone level, 1.58 ng/mL; and, estradiol level, 32.84 pg/mL. No significant sign of infection or internal bleeding was indicated by the laboratory data. There were no significantly increased tumor markers, such as the carbohydrate antigen 125 level (20.2 IU/mL), carbohydrate antigen 19–9 level (10.20 IU/mL), and carcinoembryonic antigen level (0.649 ng/mL). The chest X-ray showed nodular densities in the right lower lung (Figure 2).

**Figure 3:**
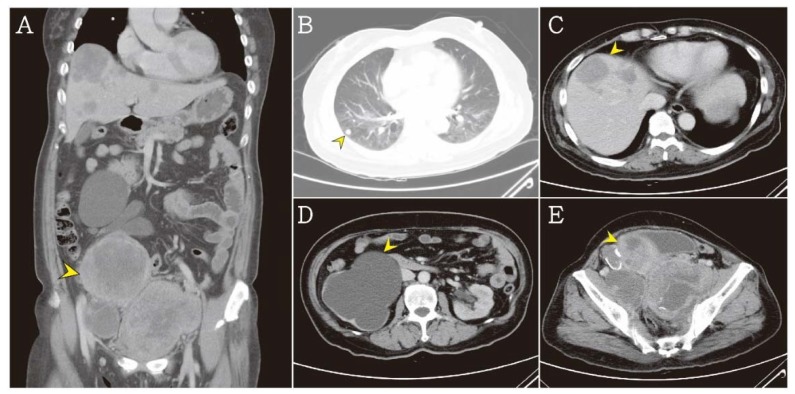
(**A**) The abdominal computed tomography revealed a huge heterogeneous density mass (arrow head). (**B**) Multiple pulmonary nodules were noted in lung field (arrow head). (**C**) Multiple heterogeneous hepatic tumors were noted (arrow head). (**D**) Severe hydronephrosis at right kidney were noted due to tumor compression (arrow head). (**E**) Three parts of heterogeneous pelvic tumors were noted about 9.4 × 8.6 × 9.3 cm^3^, 7.1 × 6.1 × 6.0 cm^3^, 11.1 × 7.5 × 9.1 cm^3^ (arrow head).

Contrast-enhanced whole-body computed tomography was performed and revealed three heterogeneous pelvic tumors, which were measuring approximately 9.4 × 8.6 × 9.3 cm^3^, 7.1 × 6.1 × 6.0 cm^3^, and 11.1 × 7.5 × 9.1 cm^3^, with multiple pulmonary nodules and multiple heterogeneous hepatic tumors. Therefore, distant metastasis was suspected. Severe hydronephrosis of the right kidney was obvious due to tumor compression (Figure 3A–E).

**Figure 4:**
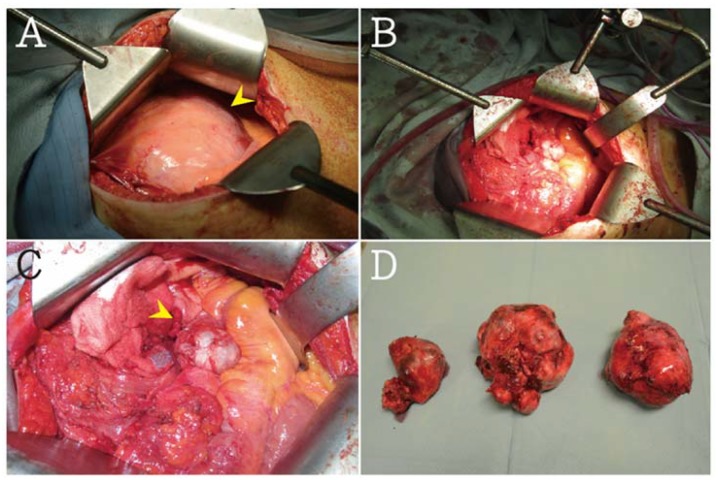
(**A**) In exploratory laparotomy, a huge mass about 14.5 × 12 × 9 cm^3^ and totally 1400 g was noted (arrow head). (**B**,**C**) Right ovary was found in right pelvic cavity and adhesive to interbowel loops (arrow head). (**D**) Three parts of heterogeneous pelvic tumors were removed (arrow head).

Exploratory laparotomy was recommended and performed. Three individual bulky tumors were encountered in the retroperitoneal pelvic cavity, which were found by the direct compression of the urinary bladder and both ureters. A solitary tumor mass measuring about 14.5 × 12 × 9 cm^3^ and weighing 1400 g was excised from the left paracolic gutter with extensive abdominal peritoneal carcinomatosis. The smooth-surfaced tumor seedings were suspected to be benign myomatous lesions. The right ovary was found in the right pelvic cavity and adhered to the interbowel loops. The pelvic tumor partially invaded the urinary bladder (Figure 4A–D). Nearly complete cytoreductive surgery was performed. The three individual bulky tumors were resected with the left ovary, right adnexa, paracolic gutter tumor, and cul-de-sac cells for cytology. Unlike complete staging surgery, she did not undergo omentectomy, appendectomy, and lymph node assessment.

Results of the final pathology report showed that the largest encapsulated tumor had many spindle cells with multinucleated pleomorphic nuclei, extensive necrosis, and a mitotic index of 11/10 per high power field, which is consistent with high-grade leiomyosarcoma (differentiation score = 3, mitotic score = 2, necrotic score = 2, total score = 7; French Federation of Cancer Centers Sarcoma Group histologic grade 3). Immunohistochemistry staining showed that the tumor cells were reactive with desmin, negative with cytokeratin and CD117 (c-kit), and negative for estrogen and progesterone receptors. The immunohistochemical profile confirmed leiomyomatous differentiation in other small nodules that were seeding in the peritoneum and omentum, smaller pelvic masses with hypercellular spindle cell tumors, and minimal nuclear atypia, which were all indicative of leiomyomatosis. She was diagnosed as having a leiomyosarcoma arising from DPL at 61 years of age. Chemotherapy with doxorubicin caused the leiomyosarcoma.

Unfortunately, she died approximately one month after the diagnosis because of rapid progression of pleural effusion due to malignancy.

## 3. Discussion

Benign smooth muscle cells are mainly involved in DPL. Because of its rare prevalence rate, the origin of DPL remains unclear. A couple studies have hypothesized that DPL may arise from submesothelial multipotential cells with multiple factor stimuli for cell differentiation, such as hormones and genes [13,14]. The association between DPL and endometriosis or leiomyomas has been investigated. In previous studies, the metaplastic phenomenon and mullerianosis may involve the mechanism of DPL formation [14,15,16,17,18,19]. Generally, patients with DPL have a good prognosis, but the malignant transformation of DPL has been rarely reported. In addition, the coexistence of DPL and leiomyosarcoma has been reported in only a few case reports. In 2016, Tun et al. [11] reported a rare case of DPL that is concomitant with a leiomyosarcoma of the pelvis in a 56-year-old woman with a history of leiomyoma of the uterus. Her prognosis was poor because of the rapid progression of malignancy.

The detailed mechanism of malignant transformation of DPL plays an important role in the ability of physicians to detect the progression of malignancy early and to provide timely intervention. Currently, a new concept of epithelial-to-mesenchymal transition (EMT) suggests that malignant transformation is induced by EMT-activating transcription factors, which regulate all stages of cancer progression, including tumor proliferation, invasion, migration, distant metastasis, and drug resistance [20,21]. In mesenchymal tumors, such as bone and soft tissue sarcomas, the involvement of EMT is still largely unclear; however, several studies revealed that many sarcomas can progress by EMT-related processes and may manifest mesenchymal-to-epithelial transition [21]. In 2017, Yang et al. [22] reported a mesenchymal-to-epithelial reverting transition in leiomyosarcoma through the regulation of the expression of E-cadherin and the E-cadherin repressor, Slug/SNAI2. The EMT-involved tumor manifested with an advanced clinical course due to higher activity of tumor proliferation, invasion, metastasis, and drug resistance. In our case, the patient had a history of recurrent DPL for 21 years without any evidence of malignancy. During the long-term follow-up, acute progression of the mass induced multiple distant metastases within only three years. The pathological examination confirmed the diagnosis of leiomyomatous differentiation with multinucleated pleomorphic nuclei, extensive necrosis, and a higher mitotic index. On the basis of our patient’s advanced clinical course, we suspected that EMT was involved, leading to the rapid malignant transformation from a benign tumor to a leiomyosarcoma. We think that it is necessary for clinicians to perform molecular and cytogenetic analyses of DPL as routine analyses in order to detect EMT-related processes and the malignant transformation of DPL.

In previous clinical reports, DPL with malignant transformation was rarely discussed. In Table 1, we present previously reported cases and summarize their clinical features, therapies, and prognoses. In 2004, Sharma et al. [10] reported an unusual occurrence of DPL with malignant transformation to a leiomyosarcoma in a 55-year-old post-menopausal woman. However, her previous records of surgery and subsequent histopathologic evaluation were missing. In 2015, Żyła et al. [23] described a 26-year-old woman with short-term recurrent abdominal pain and vaginal bleeding after removal of a leiomyoma. The imaging study revealed DPL, but the detailed diagnostic procedures were not performed. After two years, a pelvic mass was observed and removed. Results of the pathology report showed a low-grade non-metastatic endometrial sarcoma with minimal pleomorphism and a moderate mitotic index (6/10 per high power field). The patient’s clinical outcome was good. When compared to previous data, our patient had a longer follow-up duration and underwent more surgeries for recurrent DPL. However, results of the pathology report indicated no significant evidence of malignancy. Results of the laboratory evaluation of hormonal and tumor markers were also unremarkable. Further, B symptoms, such as a fever, night sweats, and weight loss, were not present in our patient, and no significant vaginal bleeding or discharge was noted. Results of a previous Pap smear revealed only inflammation without atypical cells. Panendoscopy showed only a mild gastric and duodenal ulcer with no evidence of malignancy. Thus, we conclude that no distinct clinical features are predictive of malignancy. Based on reported cases, young age and the early detection of malignancy may improve patients’ clinical outcome before rapid progression of multiple metastases.

Our patient was diagnosed as having DPL at 61 years of age. She underwent nearly complete staging surgery. In previous studies, the surgery performed was based on the tumor size. The huge tumor was resected as complete staging surgery because we suspected malignancy. In our patient, we think that the several instances of recurrence were an indicator of malignancy and the need for advanced therapies. Atypia in the pathology report may have been another indicator of early malignant transformation. Therefore, we think that molecular and cytogenetic analyses of DPL should be routine. Herein, we highlighted the clinical and imaging features of leiomyosarcoma so that physicians can make an early diagnosis and provide timely medical and surgical intervention to improve outcomes. We also recommend that these rare cases be referred to specialized centers.

## Figures and Tables

**Table 1:** A summary of previously reported cases of disseminated peritoneal leiomyomatosis with malignant transformation in women.

Study	Age	Obstetrical History	Clinical Presentation	OC Use	Location	MT Interval	Malignancy	Surgery	Adjuvant Therapy	Outcome
Rubin et al. [4]	27	G1	Found at cesarean section	ND	1. Pelvis 2. Bone metastases	6 months	Small spindle cell sarcoma	1. TAH and RSO 2. Partial omentectomy 3. Resection of peritoneal implants	Radiotherapy Chemotherapy: 1. Doxorubicin 2. Cyclophosphamide 3. Cisplatin	Died 23 months
Akkersdijk et al. [3]	25	G0	Acute right lower abdominal pain.	None	1. Omentum 2. Colon 3. Small intestine	1 year	LMS	1. TAH and RSO 2. Partial omentectomy 3. Resection of peritoneal implants	Hormonal therapy: 1. GnRH agonists	Died 22 months
Abulafia et al. [6]	20	G0	Left lower abdominal pain for 2 months	None	1. Omentum 2. Pelvis	1 year	LMS	1. Cytoreductive surgery 2. Omentectomy	Hormonal therapy: 1. Leuprolide acetate Chemotherapy: 1. Doxorubicine 2. Ifosfamide 3. Etoposide	Died 24 months
Raspagliesi et al. [5]	26	G0	ND	None	1. Left adnexa	4 months	LMS	1. Adnexectomy	Chemotherapy: 1. Infliximab 2. Dacarbazine 3. Epirubicin	No recurrences after 3 years
Fulcher AS et al. [7]	48	G2	Dyspareunia and severe pelvic pain	None	1. Pelvis 2. Subdiaphragmatic mass	3 months	LMS	1. TAH and BSO 2. Omentectomy 3. Resection of peritoneal nodules 4. Biopsy of subdiaphragmatic mass	Chemotherapy: 1. Epirubicin 2. Ifosfamide 3. Dacarbazine	Died
Morizaki et al. [9]	33	G0	Lower abdominal pain	None	1. Peritoneum 2. Mesentery 3. Descending colon 4. Pelvis	4 months	1. LMS 2. Fibrosarcoma	1. Laparotomy 2. Resection of peritoneal nodules	Chemotherapy: 1. Cisplatin 2. Cyclophosphamide	Died 11 months
Sharma et al. [10]	55	ND	Abdominal swelling for 1 year	None	1. Omentum 2. Mesentery	1 year	LMS	1. Laparotomy 2. Resection of peritoneal nodules	No	ND
Lamarca et al. [24]	37	G0	Increased abdominal perimeter	None	1. Peritoneal cavity	0 month	LMS	1. TAH and BSO 2. Tumor mass resection	Hormonal therapy: 1. Triptorelin Chemotherapy: 1. Adriamicine 2. Gemcitabine 3. Isophosphamide 4. Trabectedin 5. Sorafenib	Died 24 months
Zyla MM et al. [23]	26	G0	Abdominal pain and vaginal bleeding	OCP	1. Omentum 2. Peritoneal cavity 3. Pelvis 4. Retroperitoneal space	1 year	Endometrial sarcoma	1. TAH and BSO 2. Omentectomy 3. Appendectomy 4. Pelvic lymphadenectomy 5. Tumoral mass resection	Hormonal therapy: 1. GnRH agonists	No recurrences after 1 year
Tun AM et al. [11]	56	ND	Abdominal distension	ND	1. Pelvis 2. Peritoneum 3. Omentum 4. Lung and liver metastases	ND	LMS	1. Exploratory laparotomy 2. Debulking of the tumors	Chemotherapy: 1. Gemcitabine 2. Docetaxel	Died 5 months
Shahin NA et al. [12]	48	ND	Menorrhagia for one year	None	1. Right lumbar 2. Pelvis 3. Left upper abdomen 4. Douglas pouch	6 months	LMS	1. Laparotomy 2. Resection of the mass	Chemotherapy	Died 15 months
Syed M et al. [12]	40	ND	Abdominal pain for 7 months	OCP	1. Peritoneum 2. Bilateral adnexa 3. Recto-uterine pouch 4. Pre-vesical space 5. Left rectus 6. Abdominis muscle	3 years	LMS	1. TAH and BSO 2. Debulking of the tumors	ND	ND

LMS: leiomyosarcoma, ND: no data, OC: oral contraceptives, TAH: transabdominal hysterectomy, RSO: Right side Salpingo-Oophorectomy, MT Interval: malignant transformation interval time.
